# Prediction of Lime Tolerance in *Rhododendron* Based on Herbarium Specimen and Geochemical Data

**DOI:** 10.3389/fpls.2018.01538

**Published:** 2018-10-23

**Authors:** Shusheng Wang, Leen Leus, Marie-Christine Van Labeke, Johan Van Huylenbroeck

**Affiliations:** ^1^Plant Sciences Unit, Applied Genetics and Breeding, Flanders Research Institute for Agriculture, Fisheries and Food (ILVO), Melle, Belgium; ^2^Department of Plants and Crops, Faculty of Bioscience Engineering, Ghent University, Ghent, Belgium; ^3^Lushan Botanical Garden, Jiangxi Province and Chinese Academy of Sciences, Jiujiang, China

**Keywords:** *Rhododendron*, lime tolerance, pH, calcium carbonate, herbarium specimen, geocoding

## Abstract

Rhododendrons are typically known to be calcifuges that cannot grow well in lime soils. Data on lime tolerance of different taxa in *Rhododendron* are scarce. Habitats of naturally distributed specimens of genus *Rhododendron* were compiled as Chinese text-based locations from the Chinese Virtual Herbarium. The locations were then geocoded into latitude/longitude pairs and subsequently connected to soil characteristics including pH and CaCO_3_ from the Harmonized World Soil Database (HWSD). Using the upper quartile values of pH > 7.2 and CaCO_3_ > 2% weight in topsoil as threshold, we predicted the lime tolerant taxa. A dataset of 31,146 *Rhododendron* specimens including the information on taxonomy, GPS locations and soil parameters for both top- and subsoil was built. The majority of the specimens were distributed in soils with moderately acidic pH and without presence of CaCO_3_. 76 taxa with potential lime tolerance were predicted out of 525 taxa. The large scale data analysis based on combined data of geocoded herbarium specimens and HWSD allows identification of valuable *Rhododendron* species, subspecies or botanical varieties with potential tolerance to lime soils with higher pH. The predicted tolerant taxa are valuable resources for an in-depth evaluation of lime tolerance or for further use in horticulture and breeding.

## Introduction

The tolerance of various plant species to abiotic stresses evolves according to environmental changes in their habitats (Dimichele et al., 1987; Amtmann et al., 2005; Marais and Juenger, 2010). The evolutionary processes influenced by environmental change as well as the modern regionalization and dispersal of natural habitats have resulted in diverse biogeographical distribution patterns among different plants (Wen, 1999; Xing et al., 2015). Predicting plant species’ tolerance to abiotic stresses using distribution and geochemical data has been accepted as a potentially useful approach (Saslis-Lagoudakis et al., 2015). However, collection of large-scale distribution data of plants through field study is time-consuming. An alternative method of extracting the distribution information from the specimens in herbaria, which were collected and identified by botanists and experienced plant hunters including the location data, is effective and meaningful (Hart et al., 2014; Romeiras et al., 2014; Zhang et al., 2015).

*Rhododendron* is the largest genus in the family of Ericaceae, comprising nine subgenera, and with about 1000 species, primarily distributed in Asia, Europe, and North America. In China 571 species, 180 botanical varieties and 72 subspecies are reported (Fang et al., 2005). Although rhododendrons are of high ornamental value, they are typically recognized as calcifugous plants (which cannot grow well on lime/calcareous soils), and they usually grow well in soils with pH of 4.5 to 6.0 (Kinsman, 1999). Rhododendrons growing in pH-neutral or alkaline soils frequently suffer from iron (Fe) deficiency chlorosis symptoms: interveinal chlorosis in newly formed leaves, shoot and root growth reduction, leaf wilting, defoliation, and finally, plant death (Demasi et al., 2015a). Fe deficiency may be caused by physical or chemical properties of lime soils, which contain high bicarbonate (HCO_3_^-^) concentrations in their soil solution (Mengel, 1994). Mordhorst et al. (1993) found that high calcium cation ([Ca^2+^]) supply (in absence of HCO_3_^-^) does not suppress growth in *Rhododendron*. Other researchers found that the influence of calcium compounds on the development of *Rhododendron* micro-cuttings did not depend on the amount of assimilated Ca^2+^ ions but rather on the type of anions present in given salts (Giel and Bojarczuk, 2002). Further work showed that the major factor limiting *Rhododendron* growth in calcareous soils is the increase in substrate pH, rather than an increase in the concentration of calcium ions (Giel and Bojarczuk, 2011).

The Chinese Virtual Herbarium (CVH^1^), an online portal allowing access to herbarium specimen information, is a collaboration of more than 30 major herbaria in China and consists of more than 6 million specimens. CVH lists about 90 thousand *Rhododendron* specimens. However, the information on the collection locations of the specimens are mostly recorded in Chinese text instead of Global Position System (GPS) data due to the absence of portable precise positioning devices at the time of collection. Therefore, this information cannot be used directly for visualization of plant distribution and it hampers the automatic connection with other databases such as the Harmonized World Soil Database (HSWD). The HSWD is a 30 arc-second raster database with over 15,000 different soil mapping units that combines existing regional and national updates of soil information worldwide (Batjes et al., 2012).

Geocoding is often described as the process of converting text-based address data into digital geographic coordinates, most commonly resulting in latitude/longitude pairs (Goldberg, 2011). Geocoding technology is increasingly important in the coming era of big data to bridge the gap between non-spatial and spatial data in various fields, such as epidemiology (Bergman et al., 2012; Nuvolone et al., 2016), environmental health (Faure et al., 2017), land or forest economy (Johnston et al., 2016; Moeltner et al., 2017), and so on. Because of its great importance, many geocoding methods have been developed including online services, commercial in-house services, as well as no-cost strategies using R (Goldstein et al., 2014; Faure et al., 2017). However, Chinese geocoding faces great challenges due to the complexity of the address string format in Chinese, which contains no delimiters between Chinese words, and limited address reference resources (Tian et al., 2016).

In this study, the distribution map of *Rhododendron* natural taxa including species, subspecies and varieties in China were generated according to the latitude/longitude pairs geocoded from Chinese text-based addresses of herbarium specimens. We connected the obtained GPS data of the herbarium specimens with the HWSD, which enabled us to derive data on soil pH and CaCO_3_ concentrations for each specimen location. The influence of soil characteristics on the distributions of *Rhododendron* taxa and the tolerance potential of rhododendrons to high pH and CaCO_3_ concentration were evaluated. By analyzing *Rhododendron* specimens and geochemical data, we aim to illustrate and predict their potential tolerance to abiotic stress at taxon level. The large scale data analysis, which concerns a multitude of taxa and a large area, allowed us to identify valuable *Rhododendron* species, subspecies or natural varieties with tolerance potential to lime soils with higher pH.

## Materials and Methods

### Data Collection of Herbarium Specimens and Geocoding

In this study, *Rhododendron* species, subspecies, and botanical varieties were regarded as independent taxa (taxonomic units). Data on *Rhododendron* taxa were collected from CVH. Approximately 90,000 *Rhododendron* specimens were present in that database. Taxonomic data as well as the Chinese text-based locations of herbarium specimens were extracted in R (R Development Core Team^2^), using packages “RCurl” (version 1.95-4.8) and “XML” (version 3.98-1.5). The Latin names and subgenus information were subsequently revised and uniformed according to *Flora of China* (Fang et al., 2005).

The Chinese text-based locations of each specimen were geocoded to GPS latitude/longitude pairs using the R package “REmap” (Lang, 2016) based on Baidu Maps API (Application Program Interface).

### Soil Parameters and Data Cleaning

The Harmonized World Soil Database (HWSD, version 1.21) (Batjes et al., 2012) was used to obtain the soil data information. “MU_GLOBAL” (Global Mapping Unit Identifier) provided the link between the Geographic Information System (GIS) layer and the attribute database. Mainland China can be recognized by “MU_GLOBAL” from 11000 to 11935. For each of these 936 mapping units, there is only one set of soil data with physical and chemical parameters. First “MU_GLOBAL” of the specimens with obtained latitude/longitude pairs were extracted in ArcGIS 10.2 (ESRI, Redlands, CA, United States) based on HWSD projected in spatial reference of WGS_1984 at a resolution of 0.0083 decimal degrees, which covered a grid cell of about 1 km × 1 km. Then soil parameters, including pH measured in a soil-water solution and CaCO_3_ of both topsoils (0 ∼ 30 cm) and subsoils (30 ∼ 100 cm) were obtained by connecting the specimen location data and HWSD data using the same “MU_GLOBAL”.

The specimens from which the calculated location was linked via “MU_GLOBAL” to areas identified as either ‘inland water,’ ‘rock debris,’ ‘glaciers and permanent snow,’ or ‘urban area’ were removed from the soil parameters dataset. These areas are indicated by the symbols “WR,” “RK,” “GG,” or “UR,” respectively, under the soil unit symbol “SU_SYM90”. Specimens lacking subsoil data were also deleted.

### Data Analysis

Accuracy of the geocoding algorithm was evaluated by calculating the distance between the automated geocoded locations and the original field-recorded locations by “distVincentyEllipsoid()” in R package “geosphere” (Hijmans, 2017). The number of specimens for each taxon were counted and taxa were classified according to the nine subgenera of *Rhododendron* listed in *Flora of China* (Fang et al., 2005). Heat maps (Kernel Density) were generated in ArcGIS 10.2 to visualize the distribution centers of the genus *Rhododendron*. The number and percentage of grid cells, specimens and taxa were calculated according to the different pH and CaCO_3_ ranges for both top- and subsoil. For pH, five ranges (<4.5, 4.5–5.5, 5.5–7.2, 7.2–8.5, and >8.5) and for CaCO_3_ (% weight), four classes (<2, 2–5, 5–15, and >15), were defined according to HWSD. For each taxon with at least 10 specimens in our database, we determined lower quartile (LQ), median and upper quartile (UQ) for both pH and CaCO_3_ of topsoil. Median values hereby provide the taxon’s central tendency to environmental conditions (pH and CaCO_3_) in their distributions, while LQ and UQ values represent more extreme conditions that rhododendrons counter within their habitats. Because we aimed to predict the tolerance potential of rhododendrons to lime soils with high pH and CaCO_3_ concentration, we considered the UQ as tolerance indicator. The taxa with UQ of pH > 7.2 and CaCO_3_ > 2% weight in topsoils were predicted as being potentially tolerant to lime soils. To evaluate the results of prediction, the number of specimens distributed in lime (pH > 7.2 and CaCO_3_ > 2%) or non-lime (pH ≤ 7.2 and CaCO_3_ ≤ 2%) top soils of predicted tolerant and non-tolerant taxa were calculated, a Chi-square test in R was then performed. The distribution of the taxa as predicted in this study and based on literature reports of taxa with lime tolerance potential was mapped using ArcGIS 10.2.

## Results

### Database Construction

Of the nearly 90,000 *Rhododendron* specimens present in the CVH, 69,129 specimens containing Chinese text-based locations could be collected. Of those, the locations of 35,574 specimens were geocoded to GPS latitude/longitude pairs with a labeled text that best matched the location text of specimen. As a vast majority of specimens didn’t include the field-recorded GPS locations, we randomly selected 60 specimens with GPS information which could be found from the scanned picture of specimens in CVH. The average distance between the automated geocoded locations and the field-recorded locations was 31.2 ± 7.5 km (1st quantile 3.7 km, median 7.0 km, 3rd quantile 22.2 km). From these 35,574 specimens, the “MU_GLOBAL” data were extracted based on specimen GPS locations and HWSD. The obtained data showed that “MU_GLOBAL” of 382 specimens were located outside of mainland China. Furthermore, 458 specimens were located in ‘inland water,’ ‘rock debris,’ ‘glaciers and permanent snow,’ or ‘urban area’ and 3,588 specimens lacked subsoil data. All of these specimens were discarded from the dataset. Finally, we built a database of 31,146 specimens including the information on taxonomy, GPS locations and soil parameters for both top- and subsoil. This database was used for subsequent data analysis.

### *Rhododendron* Taxa and Distribution

**FIGURE 1:**
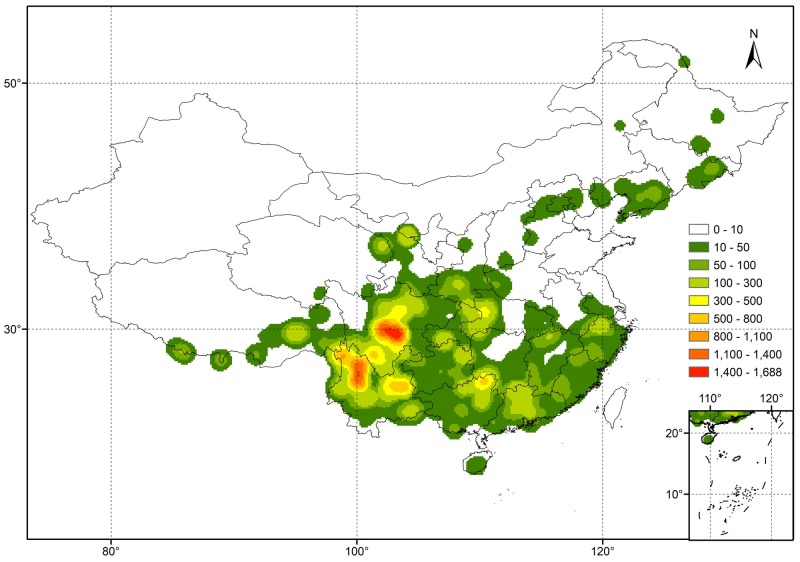
Heat map (Kernel Density) showing the distribution in China of the 31,146 *Rhododendron* specimens present in our dataset.

The 31,146 specimens of our dataset were divided into 413 species, 78 varieties and 34 subspecies. The species represented 72.3% of the described species in China and covered all nine subgenera (Table 1), while varieties and subspecies represented 43.3 and 47.2% of the described Chinese *Rhododendron* varieties and subspecies, respectively. For subsequent analyses the varieties and subspecies were treated at the same level as species as separate taxa: in total, 525 taxa were analyzed. The number of specimens for each taxon varied from 1 to 1435 (Supplementary Table S1). For 302 of these, at least 10 specimens were present, while 37 had more than 200 specimens. *Rhododendron simsii*, one of the most common species in genus *Rhododendron*, has the most specimens, followed by *R. decorum*, *R. micranthum*, and *R. mariesii*, etc. The 20 species with most specimens represented 36.8% of the complete database (Table 2). The heat map made from the 31,146 specimens showed that genetic resources of genus *Rhododendron* were mainly distributed in southwest China, with two hotspots in Sichuan and Yunnan province (Figure 1).

**Table 1:** Distribution of the different species present in the database in nine subgenera of *Rhododendron*.

Subgenus of *Rhododendron*	Number of species present in our dataset^z^	Number of described species in China^y^
*Rhododendron*	137	184
*Pseudazalea*	3	6
*Pseudorhodorastrum*	8	10
*Rhodorastrum*	2	2
*Hymenanthes*	181	259
*Azaleastrum*	18	26
*Pentanthera*	2	2
*Tsutsusi*	61	81
*Therorhodion*	1	1
Total	413	571


*^*z*^The number of collected species showed here excludes the 78 botanical varieties and 34 subspecies.*

*^*y*^Based on Fang et al. (2005) and also excluding the varieties and subspecies.*

**Table 2:** List of top 20 *Rhododendron* species represented by the highest numbers of specimens.

Latin name	Number of specimens
*Rhododendron simsii*	1435
*Rhododendron decorum*	1374
*Rhododendron micranthum*	826
*Rhododendron mariesii*	799
*Rhododendron racemosum*	630
*Rhododendron oreodoxa*	582
*Rhododendron stamineum*	573
*Rhododendron ovatum*	556
*Rhododendron rubiginosum*	488
*Rhododendron pachytrichum*	457
*Rhododendron yunnanense*	447
*Rhododendron mariae*	431
*Rhododendron concinnum*	391
*Rhododendron microphyton*	378
*Rhododendron augustinii*	376
*Rhododendron vernicosum*	374
*Rhododendron siderophyllum*	357
*Rhododendron irroratum*	342
*Rhododendron mucronulatum*	322
*Rhododendron lutescens*	320


### Effect of Soil pH

**FIGURE 2:**
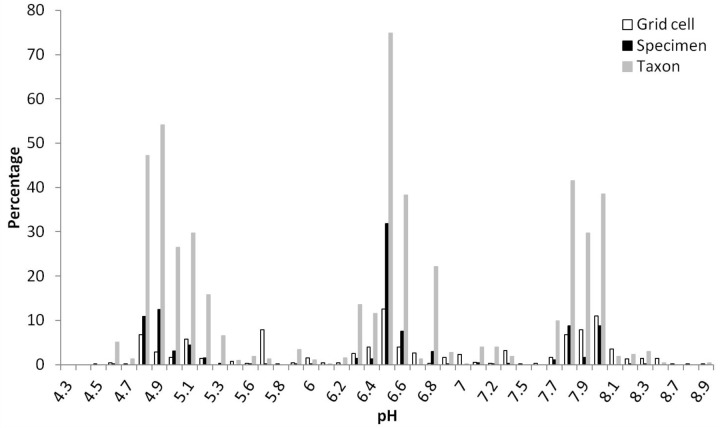
Percentages of grid cells (10,804,183 in total), *Rhododendron* specimens (31,146 in total) and taxa (525 in total) in different pH values for topsoil (pH measured in a soil-water solution and different specimens of one taxon could be found at diverse pH values).

A total of 10,804,183 grid cells in China were mainly distributed at pH range of 5.5–7.2 (41.2%) and 7.2–8.5 (38.8%) of topsoil, with more at 7.2–8.5 (44.9%) than 5.5–7.2 (36.0%) of subsoil (Table 3). More than 75% of *Rhododendron* specimens were located in areas with top- and subsoil pH ranging between 4.5 and 7.2 (Table 3). About 20% of the specimens were linked to a topsoil pH between 7.2 and 8.5. Almost no specimens or taxa were distributed in areas with pH lower than 4.5 or higher than 8.5. In topsoil, grid cells distributed at four humps around pH of 6.5 (12.5%), 8 (11%), 5.7 (7.9%), and 4.8 (6.7) (Figure 2). pH 6.5 grouped the most specimens (9,898, 31.8%), followed by 4.9 (3,858, 12.4%) and 4.8 (3,399, 10.9%), then 7.8 (2,735, 8.8%) and 8 (2,727, 8.8%). Similarly, most taxa (393, 74.9%) distributed in topsoil pH of 6.5, followed by 4.9 (284, 54.1%), 4.8 (248, 47.2%), 7.8 (218, 41.5%), and 8 (202, 38.5%) (Figure 2).

**Table 3:** Distribution of grid cells, *Rhododendron* specimens and corresponding taxa (525 in total) according to different pH ranges in top and subsoil.

pH(-log(H^+^))	Topsoil			Subsoil		
	Number of grid cells	Number of specimens	Number of taxa	Number of grid cells	Number of specimens	Number of taxa
<4.5	793 (0.007%)	0	0	19,041 (0.2%)	48 (0.15%)	20 (3.8%)
4.5–5.5	2,118,460 (19.6%)	10,234 (32.9%)	409 (77.9%)	1,833,929 (17.0%)	10,180 (32.7%)	404 (76.9%)
5.5–7.2	4,447,296 (41.2%)	14,442 (46.4%)	420 (80.0%)	3,886,312 (36.0%)	13,602 (43.7%)	408 (77.7%)
7.2–8.5	4,192,123 (38.8%)	6,467 (20.8%)	335 (63.8%)	4,851,365 (44.9%)	7,290 (23.4%)	340 (64.8%)
>8.5	45,511 (0.4%)	3 (0.01%)	2 (0.38%)	213,536 (2.0%)	26 (0.08%)	10 (1.90%)


### Effect of Soil CaCO_3_

**FIGURE 3:**
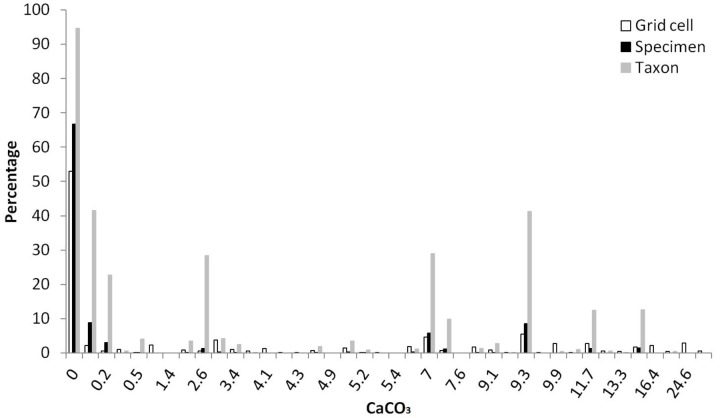
Percentages of grid cells (10,804,183 in total), *Rhododendron* specimens (31,146 in total) and taxa (525 in total) in different concentrations (% weight) of CaCO_3_ in topsoil (Different specimens of one taxon could be found at diverse CaCO_3_ concentrations).

More than a half of grid cells in China were linked with CaCO_3_ concentration less than 2% for both top (59.8%) and subsoils (52.5%), followed by concentration rang of 5–15 and 2–5 (% weight) (Table 4). A vast majority of specimens (>75%) and taxa (>95%) were distributed in top or subsoil with CaCO_3_ less than 2% weight, followed by CaCO_3_ ranging between 5–15 and 2–5 (% weight) (Table 4). Almost no *Rhododendron* taxa were distributed in soils with CaCO_3_ higher than 15% weight. In topsoils, 52.9, 5.5, 4.6, 3.8, and 2.1% of grid cells were linked with CaCO_3_ concentration of 0, 9.3, 7, 3, 0.1 (% weight) respectively (Figure 3). In topsoils without any CaCO_3_, 20,788 (66.7%) specimens and 497 (94.7%) taxa were distributed, followed by a concentration of 0.1% weight, with 2,730 (8.8%) specimens and 218 (41.5%) taxa, and 9.3% weight with 2,674 (8.6%) specimens and 217 (41.3%) taxa (Figure 3).

**Table 4:** Distribution of grid cells, *Rhododendron* specimens and taxa (525 in total) according to different CaCO_3_ ranges in top and subsoil.

CaCO_3_ (% weight)	Topsoil			Subsoil		
	Number of grid cells	Number of specimens	Number of taxa	Number of grid cells	Number of specimens	Number of taxa
<2	6,460,264 (59.8%)	24,554 (78.8%)	505 (96.2%)	5,675,678 (52.5%)	23,711 (76.1%)	503 (95.8%)
2–5	887,088 (8.2%)	724 (2.3%)	166 (31.6%)	1,107,230 (10.2%)	1,289 (4.1%)	200 (38.1%)
5–15	2,611,756 (24.2%)	5,865 (18.8%)	303 (57.7%)	3,066,378 (28.4%)	6,140 (19.7%)	304 (57.9%)
>15	845,075 (7.8%)	3 (0.01%)	2 (0.38%)	954,897 (8.8%)	6 (0.02%)	3 (0.57%)


### Tolerance Potential of *Rhododendron* Taxa to Lime Soils

**FIGURE 4:**
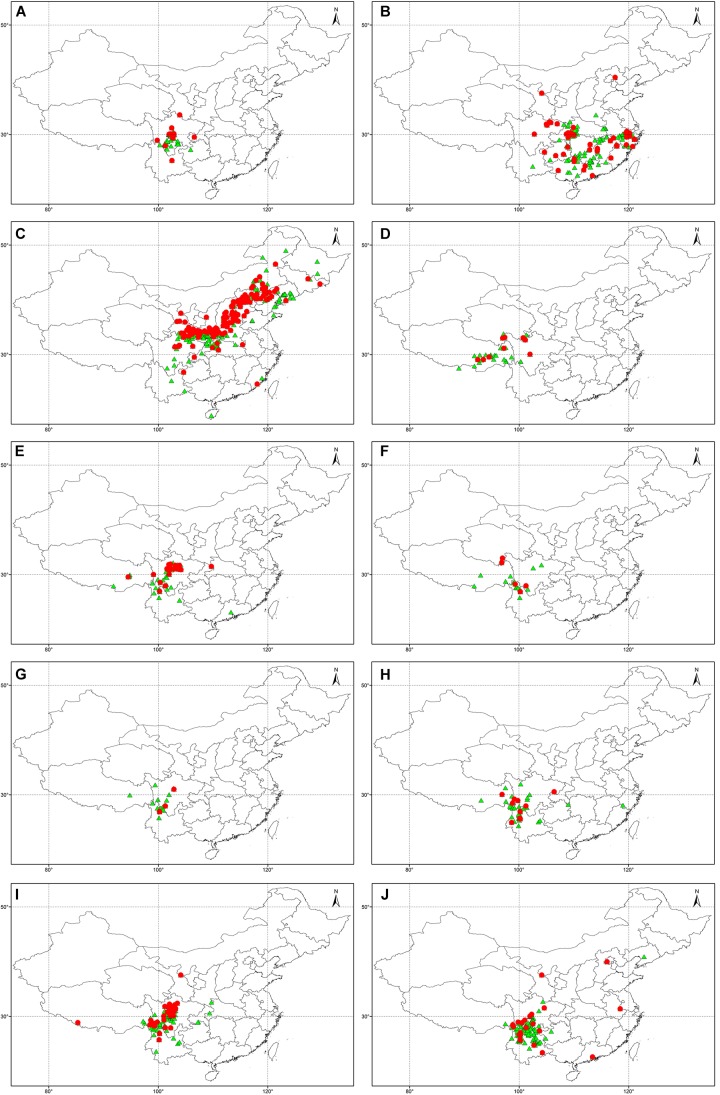
Geocoded locations of herbarium specimens from 10 *Rhododendron* taxa predicted in this study and supported by literature as being lime tolerant: *R. davidsonianum* **(A)**, *R. fortunei* **(B)**, *R. micranthum* **(C)**, *R. nivale* **(D)**, *R. phaeochrysum* **(E)**, *R. primuliflorum* **(F)**, *R. telmateium* **(G)**, *R. trichocladum* **(H)**, *R. vernicosum* **(I)**, *R. yunnanense* **(J)**. 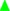: Specimen at a location with pH < 7.2 and CaCO_3_ content < 2% weight in topsoils. 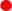: Specimen at a location with pH > 7.2 and CaCO_3_ content > 2% weight in topsoil.

Using the UQ values of pH > 7.2 and CaCO_3_ > 2% weight in topsoil as a threshold, 76 *Rhododendron* taxa were identified as potentially tolerant to lime soils, which are characterized by a higher pH and higher CaCO_3_ content (Table 5). The different taxa belong to the nine *Rhododendron* subgenera. In the predicted tolerant taxa, 2,995 and 5,443 specimens were distributed in lime and non-lime soils, respectively. And in the predicted non-tolerant taxa, 3,475 and 19,111 specimens were distributed in lime and non-lime soils, respectively. The Chi-square test showed that the distribution of specimens in lime and non-lime soils was significant different between predicted tolerant and non-tolerant taxa (*X*^2^ was 1503.7, df = 1 and *p*-value < 2.2 × 10^-16^). Using our geocoding method, we observed that in 20 taxa, more than half of the specimens were located in soils with pH > 7.2; for 53 taxa, at least one-third of the specimens were located in soils with topsoil pH > 7.2. For 10 taxa, whose tolerance potential was also supported by literature (*R. davidsonianum*, *R. fortunei*, *R. micranthum*, *R. nivale*, *R. phaeochrysum*, *R. primuliflorum*, *R. telmateium*, *R. trichocladum*, *R. vernicosum*, *R. yunnanense*), the geocoded locations of the specimens growing in topsoil with pH > 7.2 and CaCO_3_ > 2% weight were compared with the location of the other specimens (Figure 4).

**Table 5:** Lower quartile (LQ), median and upper quartile (UQ) values of pH and CaCO_3_ in topsoils and specimen distribution for the 76 taxa with UQ values of topsoil pH > 7.2, CaCO_3_ > 2% weight and a minimum of 10 specimens.

				Topsoil pH						Topsoil CaCO_3_ (% weight)				
Latin name	Sub-genus^∗^	Total number of specimens	LQ	Median	UQ	% of specimens			LQ	Median	UQ	% of specimens		
						4.5 ∼ 5.5	5.5 ∼ 7.2	7.2 ∼ 8.5				<2	2 ∼ 5	5 ∼ 15
*R. aganniphum* var. *flavorufum*	5	16	6.55	6.6	8	12.5	50	37.5	0.05	0.1	7	62.5	0	37.5
*R. alutaceum*	5	10	6.4	6.45	8	20	40	40	0	0	7	60	0	40
*R. amesiae*	1	13	5.1	6.5	8	38.5	15.4	46.2	0	0	7	53.8	7.7	38.5
*R. anthopogonoides*	1	112	7.1	7.3	7.8	0	39.3	60.7	2.6	4.6	5.5	23.2	51.8	25
*R. balfourianum* var. *aganniphoides*	5	23	6.5	6.6	8	4.3	56.5	39.1	0	0.1	7	60.9	4.3	34.8
*R. brachyanthum*	1	10	6.6	7.8	7.8	10	20	70	0.1	9.3	9.3	30	0	70
*R. bracteatum*	1	50	6.5	7.8	8	2	46	52	0	7	7	48	0	52
*R. bulu*	1	17	6.5	6.5	7.7	17.6	47.1	35.3	0	0	7.4	64.7	5.9	29.4
*R. capitatum*	1	90	7.3	7.3	7.8	1.1	20	78.9	4.6	5	6	12.2	42.2	45.6
*R. chrysodoron*	1	42	4.9	6	7.8	26.2	45.2	28.6	0	0	9.3	71.4	0	28.6
*R. concinnum*	1	391	6.5	6.5	7.8	15.1	55.5	29.4	0	0	7	70.6	0	29.4
*R. crassimedium*	8	12	4.9	7.15	7.8	33.3	16.7	50	0	4.7	9.3	50	0	50
*R. crinigerum*	5	67	6.5	6.8	8	16.4	38.8	44.8	0	0.2	7	55.2	1.5	43.3
*R. dauricum*	4	242	5.7	6.4	7.8	0.8	69	30.2	0	0	3.4	69.8	11.2	19
*R. davidii*	5	126	6.5	6.5	7.8	23	46.8	30.2	0	0	7	69.8	1.6	28.6
*R. davidsonianum*	1	316	6.5	6.5	7.7	15.8	57.6	26.6	0	0	7	73.4	0	26.6
*R. excellens*	1	56	4.8	5.75	7.8	50	23.2	26.8	0	0	5.95	73.2	1.8	25
*R. fastigiatum*	1	134	6.5	6.6	7.8	20.9	47	32.1	0	0.1	7	67.9	3.7	28.4
*R. flavidum*	1	33	6.5	6.5	8	9.1	51.5	39.4	0	0	7	60.6	3	36.4
*R. fortunei*	5	319	4.9	6.5	7.8	44.2	29.5	26.3	0	0	2.6	73.7	1.9	24.5
*R. hanceanum*	1	78	4.9	4.9	7.8	65.4	3.8	30.8	0	0	9.3	69.2	0	30.8
*R. hemitrichotum*	3	28	7.2	8	8	0	25	75	1.3	7	7	25	10.7	64.3
*R. henryi*	6	115	4.8	5	7.8	63.5	1.7	34.8	0	0	9.3	65.2	0.9	33.9
*R. henryi* var. *dunnii*	6	19	4.8	4.9	7.8	57.9	0	42.1	0	0	9.3	57.9	0	42.1
*R. hongkongense*	6	18	5	7.8	7.8	44.4	0	55.6	0	9.3	9.3	44.4	0	55.6
*R. hunnewellianum*	5	36	6.5	6.55	7.8	13.9	44.4	41.7	0	0	9.3	58.3	5.6	36.1
*R. impeditum*	1	17	6.5	6.6	7.9	17.6	41.2	41.2	0	0.1	7	58.8	5.9	35.3
*R. intricatum*	1	69	6.5	6.5	8	2.9	56.5	40.6	0	0	7	59.4	1.4	39.1
*R. kongboense*	1	13	6.5	6.5	8	0	53.8	46.2	0	0	7	53.8	0	46.2
*R. longesquamatum*	5	38	6.5	6.5	7.8	15.8	57.9	26.3	0	0	2.6	73.7	2.6	23.7
*R. mainlingense*	1	10	6.5	6.5	7.7	10	60	30	0	0	7.4	70	0	30
*R. micranthum*	1	826	6.5	6.6	8	5.3	51.3	43.3	0	0.1	7	54.7	6.3	39
*R. molle*	7	179	4.9	5.9	7.8	44.1	28.5	27.4	0	0	2.6	72.6	3.9	23.5
*R. mucronatum*	8	46	4.9	6.6	7.8	32.6	26.1	41.3	0	0.1	9.3	56.5	0	43.5
*R. mucronulatum*	4	322	6.4	6.5	7.8	0.9	67.1	32	0	0	7.4	63.7	3.4	32.9
*R. nanpingense*	8	13	4.8	7.8	7.8	46.2	0	53.8	0	9.3	9.3	46.2	0	53.8
*R. nivale*	1	83	6.4	6.5	7.8	16.9	49.4	33.7	0	0	3.2	62.7	26.5	10.8
*R. nivale* subsp. *australe*	1	22	6.5	7.8	8	18.2	27.3	54.5	0	7	7	45.5	0	54.5
*R. nivale* subsp. *boreale*	1	55	6.5	6.5	7.85	9.1	63.6	27.3	0	0	3	72.7	3.6	23.6
*R. oreodoxa*	5	582	6.5	6.5	7.8	3.8	69.4	26.8	0	0	7	73.2	0.7	26.1
*R. oreodoxa* var. *shensiense*	5	14	6.4	6.9	7.8	14.3	42.9	42.9	0	0.1	9.3	57.1	0	42.9
*R. ovatum*	6	556	4.8	5.1	7.8	59.2	14.6	26.3	0	0	2.6	73.7	3.1	23.2
*R. phaeochrysum*	5	143	6.5	6.6	8	5.6	59.4	35	0	0.1	7	65	0	35
*R. phaeochrysum* var. *levistratum*	5	45	6.5	8	8	4.4	40	55.6	0	7	7	44.4	0	55.6
*R. praestans*	5	26	4.8	6.5	8	46.2	26.9	26.9	0	0	7	73.1	0	26.9
*R. primuliflorum*	1	69	6.5	6.5	8	24.6	47.8	27.5	0	0	5	69.6	7.2	23.2
*R. proteoides*	5	19	4.9	4.9	8	52.6	10.5	36.8	0	0	7	63.2	0	36.8
*R. przewalskii*	5	131	6.5	7.1	7.9	0.8	58	41.2	0	3	6	44.3	27.5	28.2
*R. pubescens*	3	41	5.1	6.5	7.8	34.1	34.1	31.7	0	0	7	68.3	2.4	29.3
*R. purdomii*	5	54	6.5	6.9	8	1.9	50	48.1	0	0.15	7	51.9	7.4	40.7
*R. radendum*	1	24	7.8	7.8	7.8	0	20.8	79.2	5.95	9.3	9.3	20.8	4.2	75
*R. redowskianum*	9	68	6.5	7.8	7.8	4.4	35.3	60.3	0	9.3	9.3	39.7	0	60.3
*R. rigidum*	1	29	6.5	6.6	8	0	55.2	44.8	0	0.1	7	55.2	3.4	41.4
*R. roxieanum* var. *cucullatum*	5	22	6.8	8	8	4.5	27.3	68.2	0.2	7	7	31.8	0	68.2
*R. rufescens*	1	67	6.5	6.5	8	4.5	47.8	47.8	0	0	7	52.2	4.5	43.3
*R. rufum*	5	77	6.4	8	8	6.5	37.7	55.8	0	3	7	41.6	11.7	46.8
*R. rupicola* var. *chryseum*	1	24	6.5	7.8	7.8	16.7	29.2	54.2	0	7	9.3	45.8	0	54.2
*R. rupicola* var. *muliense*	1	23	6.5	8	8	0	47.8	52.2	0	7	7	47.8	0	52.2
*R. sanguineum* var. *himertum*	5	13	6.8	6.8	7.8	23.1	46.2	30.8	0.2	0.2	9.3	69.2	0	30.8
*R. sargentianum*	1	14	6.5	6.5	7.9	21.4	35.7	42.9	0	0	2.6	57.1	28.6	14.3
*R. saxatile*	8	12	7.8	7.8	7.8	0	0	100	9.3	9.3	9.3	0	0	100
*R. sphaeroblastum*	5	43	6.5	6.5	8	9.3	44.2	46.5	0	0	7	53.5	0	46.5
*R. stamineum*	6	573	4.9	6.3	7.8	44.9	26	29.1	0	0	9.3	70.9	2.3	26.9
*R. tatsienense*	1	142	6.5	6.5	7.7	14.8	50.7	34.5	0	0	7	65.5	0	34.5
*R. telmateium*	1	58	6.5	6.5	8	17.2	56.9	25.9	0	0	7	74.1	0	25.9
*R. thymifolium*	1	77	7.1	7.3	8	1.3	36.4	62.3	3	6	7	20.8	27.3	51.9
*R. trichocladum*	2	169	6.5	6.6	8	8.9	53.8	37.3	0	0.1	7	62.7	0	37.3
*R. trichostomum*	1	135	6.5	6.5	8	2.2	60.7	37	0	0	7	62.2	2.2	35.6
*R. vellereum*	5	30	6.5	7.1	7.7	13.3	46.7	40	0	5	7.4	46.7	13.3	40
*R. vernicosum*	5	374	6.5	6.5	8	8.3	57.8	34	0	0	7	66	2.4	31.6
*R. vialii*	6	16	6.4	6.7	7.8	18.8	37.5	43.8	0	0.15	9.3	56.3	0	43.8
*R. wasonii*	5	76	6.5	6.5	7.7	14.5	44.7	40.8	0	0	7.4	59.2	1.3	39.5
*R. websterianum*	1	43	6.5	6.6	8	4.7	48.8	46.5	0	1.6	7	53.5	4.7	41.9
*R. x detonsum*	5	16	5.1	7.4	8	37.5	12.5	50	0	3.6	7	50	0	50
*R. x pulchrum*	8	28	4.8	7.8	7.8	42.9	3.6	53.6	0	5.8	9.3	46.4	3.6	50
*R. yunnanense*	1	447	6.5	6.5	7.7	21.5	52.6	26	0	0	2.6	74	1.8	24.2


*^∗^1, Rhododendron; 2, Pseudazalea; 3, Pseudorhodorastrum; 4, Rhodorastrum; 5, Hymenanthes; 6, Azaleastrum; 7, Pentanthera; 8, Tsutsusi; 9, Therorhodion.*

## Discussion

China is considered to be a center of origin for *Rhododendron*. Chinese herbaria, accessible via the CVH, conserved tens of thousands of specimens collected by botanists and plant hunters since 1884 and this number is still increasing. Using the statistical software R with package “REmap,” we geocoded 51.5% of the Chinese text-based locations into latitude/longitude pairs with an acceptable accuracy (31.2 ± 7.5 km) in terms of a continental-scale dataset. Faure et al. (2017) reported in their epidemiological study 81.4 and 84.4% of geocoded addresses using a free-online geocoding service and an in-house geocoder system, respectively. They concluded that the geocoding accuracy was higher in urban areas compared to rural areas, but comparable for the two automatic geocoding methods. The lower geocoding rate in our study might be due to the quality of the Chinese texts which described the locations. The possibility for geocoding drops when the textual description of a location is written in an irregular manner, or a location has more than one synonym which occurs among different Chinese authorities in different governmental agencies. Besides, the whole analysis relied upon data being recorded on the herbarium specimen label. If specific locality information was not recorded, no geocoding scheme could be able to resolve an accurate location. Likewise, Hart et al. (2014) excluded ∼90% of specimens in their study mostly because specific information, such as altitude and date of collection was not recorded on the herbarium specimen label. Despite availability of commercial and no-cost geocoding strategies (Goldstein et al., 2014; Faure et al., 2017), the automated geocoding of textual documents faces challenges, especially for development of language modeling methods for textual document geocoding (Faure et al., 2017). The complexity of the address string format in Chinese text-based geocoding compounds these challenges (Tian et al., 2016).

In our final extracted dataset of 31,146 specimens, the majority (72.3%) of reported Chinese *Rhododendron* taxa were present and covered all nine subgenera. This was representative of the genus *Rhododendron* in China. The heat map showed that the hotspots were mainly distributed in the Himalaya–Hengduan Mountains area in Sichuan and Yunnan province, which is consistent with previously reports of the general distribution of *Rhododendron* species in China (Milne et al., 2010; Liu et al., 2014; Yan et al., 2015). Furthermore, the heat map (Figure 1) matched well with the spatial patterns of total *Rhododendron* species richness in China estimated in 50 km × 50 km grid cells resulted from analyzing 556 *Rhododendron* species out of 571 species occurring in China by Shrestha et al. (2018). However, the hotspots still had a regional distribution, even within the Himalaya–Hengduan Mountains area. The factors that influence *Rhododendron* distribution cannot be only attributed to the climate (Kumar, 2012), soil conditions also influence *Rhododendron* growth in a specific region (Esen et al., 2004; Kamei et al., 2009). By geocoding the specimens, we were able to link specimen location to the soil characteristics of HWSD, thus leading to a metadata analysis of soil conditions for rhododendrons in nature. Rhododendrons have shallow, fibrous root systems that are restricted to the upper soil (Kinsman, 1999; Hales et al., 2009), thus our analysis focused primarily on topsoil (0–30 cm). The majority of the plants were located in topsoils with low pH (below or around 6.5), without presence of CaCO_3_. Importantly, the pH values reported in the HWSD database are soil-water pH, which is approximately 0.9 unit higher than pH in 1 M KCl (Kabala et al., 2016). The results are in accordance with the general trend that *Rhododendron* cannot grow well in neutral or alkaline soils and is considered to be a calcifugous genus (Kinsman, 1999; Demasi et al., 2015a). Kinsman (1999) reported that at almost all sites in Northwest Yunnan where *Rhododendron*s grow in shallow soils overlying limestone, the soils still had pH values of less than 6. The pH values in Kinsman’s report were also measured in soil-water solutions, but in Kinsman’s study most soil samples were taken 3–10 cm below ground surface while the topsoil pH measured in HWSD contains the 0–30 cm layer. The lower pH in topsoils (0–10 cm) can be explained by organic horizon acidification and rhizosphere interaction of plant roots (James and Riha, 1987; Wang et al., 2016).

Using data analysis, we tried to predict at taxon level (species, subspecies or varieties) which potential genetic resources might exhibit lime tolerance. Thresholds were set at UQ for pH > 7.2 and CaCO_3_ content > 2% weight. According to HWSD, pH values from 7.2 to 8.5 are indicative of carbonate rich soils which chemically form less available carbonates affecting nutrient availability (P, Fe) (Batjes et al., 2012). Further, the bioavailability of trace element cations such as copper (Cu), Zn, nickel (Ni), cadmium (Cd), and lead (Pb) and their concentration in plants is significantly reduced at pH > 7.0 (Valentinuzzi et al., 2015). In addition, calcifugous plants are intolerant to high concentrations of Ca^2+^ when combined with high pH (Vicherova et al., 2015). Using these thresholds, we compiled a list of 76 potentially lime-tolerant taxa. For 61 of these, at least 30% of the specimens were geocoded in locations from which the topsoil pH was above 7.2 and CaCO_3_ > 2% weight.

For several taxa mentioned in our prediction, support was found in literature for their lime tolerance. McAleese and Rankin (2000) showed that *R. primuliflorum* could grow in soil with pH > 7 (sampled in topsoil 10–20 cm), as well as *R. telmateium* and *R. yunnanense*, followed by *R. vernicosum*. *R. yunnanense* was also found on gravely loam soils of high pH in the Sichuan region of China, the same as *R. davidsonianum* (Reid et al., 1998). Kaisheva (2006) classified *R. phaeochrysum*, *R. balfourianum*, *R. primuliflorum*, *R. telmateium*, *R. yunnanense*, and *R. trichocladum* as lime tolerant species. *R. fortunei* was mentioned as a promising gene resource for breeding lime tolerant rhododendrons (Shujun et al., 2008). Kinsman (1999) determined *R. primuliflorum* definitely to be growing under alkaline soil conditions (pH 7.4–7.9), while *R. rupicola* var. *chryseum* and *R. proteoides*, which were also in our prediction list, were found in soil pH values below 6.

Evidence for lime tolerance can also be found in species that co-occur in similar habitats. An example is *R. nivale*, a perennial evergreen undershrub with a height of 30–120 cm, distributed in the northeastern and southeastern areas of the Tibet Autonomous Region of China, Nepal, India, Bhutan, and Sikkim (Guo et al., 2017). This species co-occurred with *R. primuliflorum* as the representative alpine species in the snowy mountains in the northwest of Yunnan (Xu et al., 1996), which grows in limestone crevices shared with *Paraquilegia anemonoides*, another species associated uniquely with limestone. This shows that *R. nivale* has a similar habitat as *R. primuliflorum*, indicating *R. nivale* may also has a good lime tolerance potential, as well as its subspecies *R. nivale* subsp. *austral* and *R. nivale* subsp. *boreale*. Moreover, *R. rupicola* was also found together with *R. primuliflorum* in limestone crevices shared with *P. anemonoides* (McAleese and Rankin, 2000). Although *R. rupicola* was included in our dataset but not predicted as a lime tolerant taxon, its two varieties *R. rupicola* var. *chryseum* and *R. rupicola* var. *muliense* were predicted with lime tolerance. The bias of geocoding or soil data of HWSD may result in the fact that *R. rupicola* was not predicted as a lime tolerant taxon in our study. It does, however, not exclude a lime tolerant potential because congeneric species often have similar ecological characteristics and use similar resources. Furthermore, similar interspecific associations can strengthen their competitive ability and promote local exclusion to non-congeneric species to obtain more living space (Yuan et al., 2018). Field and experimental work should be carried out to confirm this in the future.

In addition, some predicted lime tolerant taxa can also be supported from horticultural and physiological studies. *R. micranthum* was proven to be able to grow in containers on lime-supplemented media (McAleese and Rankin, 2000). *R.* x *pulchrum* is a well-known taxon for landscaping and breeding. It’s cultivar *R.* x *pulchrum* ‘Sen-e-oomurasaki’ showed extremely low chlorosis and mortality rates and high ferric chelate reductase activity in high pH hydroponic conditions, resulted in iron efficient genetic resources for azalea cultivation and gardening in calcareous soils (Demasi et al., 2015a,b).

This information demonstrates that our strategy to use UQ values of pH > 7.2 and CaCO_3_ > 2% weight in topsoils at the habitats of *Rhododendron* taxa can be used as an efficient indicator for prediction of lime tolerance. As a supplement to the identification of taxa where lime tolerance was already reported, our work has also identified some potentially interesting taxa for which (to our knowledge) no information is available on potential lime tolerance.

Other studies confirmed the value of herbarium specimens, especially those with detailed locations, as a source of information. Contrasting phenological responses of *Rhododendron* species to the Himalayan climate were reported by analyzing *Rhododendron* herbarium specimens located in Lijiang County, Yunnan, China (Hart et al., 2014). Elevational distribution of native orchid species compiled from CVH were investigated and the results illustrated that the elevational pattern of orchid species richness in Yunnan was collectively shaped by several mechanisms related to geometric constraints, size of the land area, and environments (Zhang et al., 2015). The research location of above two studies were at county or provincial level, the geocoded locations of *Rhododendron* specimens in our study is valuable to extract continental climate, altitude, physical and chemical data of soil, or other environmental databases with GIS layer, and extend the research area to the national level. For instance, a continent-wide dataset of occurrence records with geographical coordinates of Australian grasses were used for predicting species’ tolerance to salinity and alkalinity (Saslis-Lagoudakis et al., 2015). An increasing number of plant specimens, especially those with GPS information, have been collected in recent decades. These herbarium specimens can be used to study the plants’ tolerance to abiotic stresses, their phylogenesis, evolution, conservation efforts for endangered plants, effects of climate change or land/forest economy studies assisted by field or experimental work.

## Conclusion

Our results showed that information present in herbarium specimens might be used to identify potentially interesting genetic resources in *Rhododendron*. Geocoding of the Chinese text-based locations of plant specimens into latitude/longitude pairs makes it possible to study plant distribution as well as to connect the distribution data to soil database of the habitats. This approach makes use of a large number of plant samples, which increases the reliability of the obtained results. The combination of geocoded specimen information and the soil database led to identification of valuable resources at taxon level for tolerance against lime soils characterized by a high pH and high CaCO_3_ concentrations. The predicted tolerant taxa in this study pave the way for an in depth evaluation of potential resources for lime tolerance and in the long term for using genetic material in breeding or studies of abiotic stress. Moreover, the continental-scale dataset with both comprehensive taxonomic coverage and geocoded locations can be connected with other GIS layers such as the WorldClim database^3^ for ecological or evolutionary researches.

## Author Contributions

SW and JVH originally formulated the idea. M-CVL and LL developed methodology. SW performed statistical analyses and wrote the manuscript. All authors edited and approved the final manuscript.

## Conflict of Interest Statement

The authors declare that the research was conducted in the absence of any commercial or financial relationships that could be construed as a potential conflict of interest.

## Footnotes

1 http://www.cvh.ac.cn

2 https://www.r-project.org/

3 http://www.worldclim.org/

## Supplementary Material

TABLE S1 The number of specimens for each taxon in 525 Rhododendron taxa.
